# Fluorescence optical imaging and musculoskeletal ultrasonography in juvenile idiopathic polyarticular disease before and during antirheumatic treatment - a multicenter non-interventional diagnostic evaluation

**DOI:** 10.1186/s13075-017-1355-4

**Published:** 2017-06-30

**Authors:** Ariane Klein, Georg Werner Just, Stephanie Gabriele Werner, Prasad T. Oommen, Kirsten Minden, Ingrid Becker, Hans-Eckhard Langer, Dirk Klee, Gerd Horneff

**Affiliations:** 1 Centre for Paediatric Rheumatology, Department of Paediatrics, Asklepios Clinic Sankt Augustin, Arnold-Janssen-Strasse 29, 53757 Sankt Augustin, Germany; 2 HELIOS Klinikum Duisburg, Klinik für Rheumatologie, Duisburg, Germany; 3 Department of Pediatric Oncology, Hematology and Clinical Immunology, University Children’s Hospital, Medical Faculty, Heinrich-Heine-University, Düsseldorf, Germany; 4 Charité University Medicine and Epidemiology Unit, German Rheumatism Research Centre, Berlin, Germany; 5 Institute of Medical Statistics, University of Cologne, Cologne, Nordrhein-Westfalen Germany; 6 RHIO (Rheumatologie, Immunologie, Osteologie) Düsseldorf, Düsseldorf, Germany; 7 Department of Diagnostic and Interventional Radiology, University Düsseldorf, Medical Faculty, Moorenstraße 5, 40225 Düsseldorf, Germany

**Keywords:** Juvenile idiopathic arthritis, Imaging, Fluorescence optical imaging, Xiralite, Arthrosonography, Ultrasonography, Power Doppler

## Abstract

**Background:**

Valid detection of inflamed joints is essential for correct classification, therapeutic decisions, prognosis and assessment of treatment efficacy in juvenile idiopathic arthritis (JIA). Fluorescence optical imaging (FOI) enables visualization of inflammation in arthritis of finger and hand joints and might be used for monitoring.

**Methods:**

A 24-week observational study in polyarticular JIA patients newly starting treatment with methotrexate or an approved biologic was performed in three centers. Patients were evaluated clinically, by gray-scale ultrasonography (GSUS), power-Doppler ultrasonography (PDUS) and FOI at baseline, week 12 and week 24.

**Results:**

Of 37 patients enrolled, 24 patients started methotrexate and 13 patients a biologic for the first time (etanercept *n* = 11, adalimumab and tocilizumab *n* = 1 each). Mean JADAS 10 decreased significantly from 17.7 at baseline to 12.2 and 7.2 at week 12 and 24 respectively. PedACR 30/50/70/100 response rates at week 24 were 85%/73%/50%/27%. The total number of clinically active joints in hand and fingers at baseline/week 12/week 24 was 262 (23.6%)/162 (16.4%)/162 (9.0%). By GSUS, at baseline/week 12/week 24, 192 (19.4%)/135 (16.1%)/83 (11.5%) joints showed effusions and 186 (18.8%)/107 (12.7%)/69 (9.6%) showed synovial thickening, and by PDUS 68 (6.9%)/15 (1.8%)/36 (5%) joints showed hyperperfusion. Any sign of arthritis was detected by US in a total of 243 joints (24.5%) at baseline, 161 joints (19.2%) at week 12 and 123 joints (17%) at week 24. By FOI at baseline/week 12/week 24, 430 (38.7%)/280 (29.2%)/215 (27.6%) showed a signal enhancement in at least one phase.

Summarizing all three points of time, the highest numbers of signals were detected by FOI with 32% of joints, especially in phase 2, while by US 20.7% and by clinical examination 17.5% of joints were active. A high number of joints (21.1%) had FOI signals but were inactive by clinical examination. A total 20.1% of joints with signals in FOI did not show effusion, synovial thickening or hyperperfusion by US.

Because of the high number of negative results, specificity of FOI compared with clinical examination/US/PD was high (84–95%), and sensitivity was only moderate.

**Conclusion:**

FOI and US could detect clinical but also subclinical inflammation. FOI detected subclinical inflammation to a higher extent than US. Improvement upon treatment with either methotrexate or a biologic can be visualized by FOI and US.

**Trial registration:**

Deutsches Register Klinischer Studien DRKS00011579. Registered 10 January 2017.

## Background

Juvenile idiopathic arthritis (JIA) is the most common chronic inflammatory rheumatic disease in childhood and can lead to severe disability [1–3]. The validity of diagnosis, classification and prognosis as well as therapeutic decisions for each JIA patient depends on reliable detection of joint involvement. Careful clinical assessment is of great importance but may be difficult, especially in children. Subclinical inflammation could also be missed, for example in early stages of the disease or with minimal disease activity. Ultrasonography (US) in gray-scale (GSUS) and power Doppler (PDUS) modes is often available for assessment of synovial thickening, joint effusions and hyperperfusion, thus helping to detect active joints. In clinical practice this procedure is usually used on a limited number of joints due to limitations of time. Fluorescence optical imaging (FOI) is a contrast-enhanced optical procedure for visualization of changes in microcirculation. It has been used for over 30 years for various indications [4]. In experimental models of arthritis, indocyanine green (ICG)-enhanced FOI findings corresponded to histologically proven synovitis [5, 6]. This approach was also tested in humans [7, 8]. FOI is time efficient and operator independent. The image interpretation, however, is dependent on visual perceptions and imaging experience of the examiner and is limited to wrist and finger joints. Increased focal optical signal intensities visualize areas of high perfusion, altered microcirculation and/or capillary leakage, which may not show in GSUS, especially in the small finger joints [9–11]. In adult RA patients, FOI has been shown to be a reliable technique to detect inflammation with good agreement with magnetic resonance imaging (MRI). The authors distinguished three different phases and a computer-generated composite image; their meaning still being unclear, they may give varying information such as changes in perfusion, microcirculation, capillary leakage or neovascularization. In the opinion of the authors, very early arthritis could be observed with FOI as well as subclinical inflammation [10, 11].

FOI might be a valuable tool to assess subclinical and very early arthritis, to differentiate between arthritis and pain syndrome and to assess treatment response.

This observational study intended to analyze improvement upon newly instituted treatment with a DMARD or with a biologic clinically, using commonly used tools (PedACR, JADAS, number of active, swollen and tender joints) and by imaging methods (GSUS and PDUS) as well as by FOI. We intended for the first time to compare clinical and imaging results with the new FOI method to explore the clinical utility. Of further interest is the presentation of residual inflammation which might not be detected clinically but by imaging and whether an early decrease of inflammation detected by FOI is predictive for remission later on.

The results of the first study using the FOI technique and US examination in children with JIA before and during antirheumatic treatment are reported here.

## Methods

### Patients

In three German centers a total number of 37 patients starting a new treatment with either a DMARD or a biologic approved for polyarticular JIA were recruited for this observational study. Inclusion criteria were: diagnosis of polyarticular JIA according to ILAR criteria (seropositive, seronegative polyarthritis JIA and extended oligoarthritis) [12], active disease with at least three clinically active joints in the hand region, age 6–18 years and agreement of patient and parents/legal guardian to participate in the study. Patients had to be either naïve to methotrexate in the cohort starting methotrexate or naïve to any biologic.

The study was performed in compliance with the Declaration of Helsinki. The study protocol was approved by the ethics committee Ärztekammer Nordrhein (Number: 2012026) and the ethics committee of the Charité university clinic Berlin (Number EA2/039/12). Written informed consent forms were obtained for all participants.

### Clinical and laboratory assessment

All patients were evaluated by clinical examination (CE) at baseline, after 12 and 24 weeks. Swollen, tender joints and joints with limitation of motion (LOM) were scored for presence or absence (0–1). Joints with swelling not due to deformity or joints without swelling, LOM plus pain and/or tenderness were defined as active joints. The ESR and C-reactive protein (CRP) test were recorded at each visit. Disease activity was also measured by patient/parent visual analog scale (0–100) of overall well-being, by physician visual analog scale of overall disease activity and by CHAQ scores (0–3) [13]. The JADAS 10 score was calculated for each visit [14].

### Ultrasonography

Thirty joints were examined by ultrasonography for each patient (wrists, MCP 1–5, PIP 1–5, DIP 2–5) at three different time points (week 0, 12 and 24). Evaluation was done semiquantitatively (grades 0–3) for synovial thickening, joint effusions in GSUS and hyperperfusion in PDUS according to Manggi-Manzoni et al. [15]. Joints graded 1–3 were scored as active in the respective examination mode [10]. Ultrasonography examination was performed using LOGIQ e notebook with 12-MHz linear transducer by General Electrics Healthcare, Toshiba Aplio XG and MyLab70 XVG Esaote.

### Fluorescence optical imaging

At each visit (week 0, 12 and 24) a standardized FOI examination was performed as described by Werner et al. [10, 11]. An ICG bolus (ICG-Pulsion®, 0.1 mg/kg BW; Pulsion Medical Systems AG, Munich, Germany) was injected into the cubital vein; thereafter one image/second over 6 minutes was obtained, resulting in 360 images. Three phases according to signal intensities in the fingertips and an electronically generated composite image (CI), automatically obtained by means of the integrated software XiraView (version 3.6), were analyzed. Phase 1 includes all images until clearly increased signal intensities are visible in the fingertips, phase 2 includes all images during increased intensities in the fingertips and phase 3 includes all images from the end of phase 2 until the end of the examination. For each phase and the composite image, each joint was scored semiquantitatively (0–3: 0 = no increased signal intensity; 1 = low increased signal intensity, <25% of affected joint area; 2 = moderate intensity, >25 and <50% of affected joint area; and 3 = strong increased signal intensity, >50% of affected joint area). Joints were counted as active if they reached at least a score of 1 [10, 11]. The commercially available Xiralite X4 device (Mivenion) was used for the examination. Reading was done by a single experienced observer, who had knowledge of the typical pitfalls of FOI interpretation.

### Efficacy parameters

Clinical response was measured by the pediatric ACR (PedACR) criteria, JADAS reduction and reduction in the number of active joints. PedACR improvement was calculated as described previously in detail [16]. The categories contributing to the PedACR 30 Score are physician’s global assessment of the subject’s disease activity (numeric rating scale (NRS)), parents’ global assessment of the subject’s overall well-being (NRS), number of active joints (swelling not due to deformity or in joints without swelling, LOM plus pain and/or tenderness), number of joints with LOM, Childhood Health Assessment Questionnaire (CHAQ) and CRP. The JADAS minimal disease activity (defined as JADAS10 ≤ 3.8) and the JADAS remission activity (defined JADAS10 ≤ 1) according to the definition of Consolaro et al. [17] was calculated.

### Safety parameters

Clinical and laboratory evidence of adverse events was investigated at each study visit. The investigator assessed and recorded any adverse event in detail on the adverse event form, including the date and time of onset, description, severity, time course, duration and outcome, relationship of the adverse event to the study drug and alternative etiology for events not considered ‘probably related’ to the study drug.

### Statistical analysis

Analyses were conducted at patient and individual joint levels. Mean values and standard deviations were calculated for quantitative variables. Demographic and baseline characteristics were summarized by descriptive statistics. Efficacy and safety analyses were performed in the intention-to-treat population (ITT), and tests were two-sided. *p* < 0.05 was considered statistically significant. Frequencies were compared using the chi-square test or Fisher’s exact test as appropriate. Agreement was reached when a joint was assessed as affected (>0) or not affected (0) with both modalities. Clinical examination and ultrasonography was used as the standard reference method for calculation of sensitivity and specificity. Receiver operating characteristic (ROC) curves were generated to assess sensitivity and specificity of FOI versus ultrasonography and clinical standards of care. The area under the curve (AUC) was calculated. Improvement of continuous disease activity parameters were compared using the *t* test. Data were entered into an Access 2010 database and analyzed with Excel 2010 (Microsoft, Redmond, WA, USA) or IBM SPSS Statistics version 23.

## Results

### Study population

Thirty-seven patients with active polyarticular JIA were enrolled in this study. Of these, 24 patients started treatment with methotrexate and 13 patients received therapy with a biologic for the first time (11 patients started etanercept, one patient adalimumab and one patient tocilizumab). Thirty-four patients (21 on methotrexate and 13 on biologic) could be evaluated at week 12, and 25 patients (17 on methotrexate, eight on biologic) completed the study through week 24. Mean age at enrollment was 13.9 years (range 9–18 years).

There were no significant differences regarding patient characteristics and disease activity parameters between the two cohorts at baseline, although patients in the biologics cohort tended to have higher disease activity parameters than patients in the methotrexate cohort (Table 1).

**Table 1:** Patients’ baseline characteristics

	All patients (*n* = 37)	DMARD cohort (*n* = 24)	Biologics cohort (*n* = 13)
Male/female	7/30	5/19	2/11
Age (years)	13.9 (2.2)	14.3 (2.1)	13.5 (2.4)
Disease duration (years)	3.6 (3.2)	3.2 (4.1)	4.1 (3.7)
CHAQ-DI	0.61 (0.7)	0.54 (0.58)	0.66 (0.84)
JADAS 10	16.9 (5.7)	15.5 (5.6)	19.0 (5.0)
ESR (mm/h)	17.6 (20.1)	18.9 (21.8)	14.4 (15)
CRP(mg/l)	5.0 (12.2)	2.7 (4.5)	8.7 (18.3)
Physician Global Assessment VAS (cm)	4.8 (2.1)	4.4 (1.8)	5.5 (2.3)
Parents Global Assessment VAS (cm)	4.5 (2.5)	4.5 (2.1)	4.5 (3.2)
Number of active joints	7.1 (5.2)	6.7 (5.2)	8.3 (5.0)
Number of tender joints	9.6 (8.9)	9.6 (8.0)	10.0 (11.4)
Number of swollen joints	7.2 (5.7)	6.8 (5.7)	8.1 (5.5)
Number of joints with LOM	6.5 (6.7)	5.4 (5.3)	8.4 (8.4)

Data presented as number or mean (standard deviation)

*DMARD* disease-modifying antirheumatic drug, *CHAQ* Childhood Health Assessment Questionnaire, *CRP* C-reactive protein, *ESR* erythrocyte sedimentation rate, *JADAS* Juvenile Diseases Activity Score, *LOM* limitation of motion, *VAS* visual analog scale

Of all patients, 35% received oral corticosteroids at baseline. Also the number of patients using oral corticosteroids at baseline was slightly but insignificantly higher in the biologics cohort (seven of 13 patients) than in the methotrexate cohort (six of 24 patients). No patient had active uveitis or history of uveitis and no patient had signs or history of systemic inflammation.

### Efficacy

Response rates according to the PedACR 30/50/70/100 and the JADAS in this study were as expected. After 12 weeks of treatment, PedACR 30/50/70/100 scores were reached by 69%/45%/24%/10% in the methotrexate cohort and 82%/45%/19%/0% in the biologics cohort. Response rates improved further until week 24, with 85%/73%/50%/27% reaching PedACR 30/50/70/100 scores (83%/72%/44%/22% in the methotrexate cohort and 88%/75%/63%/38% in the biologics cohort).

All but eight patients showed decreased JADAS 10 scores at week 12, and four of these showed JADAS improvement at week 24. Mean JADAS 10 values decreased significantly during treatment for all patients. In the cohort completing week 24 of this study, the mean JADAS 10 values decreased from 17.7 at baseline to 12.2 at week 12 and 7.2 at week 24. Patients treated with DMARDs showed a decrease from 18.8 at baseline to 12.7 at week 12 and 7.6 at week 24. Patients treated with biologics started with a higher JADAS of 19.6 at baseline, but then decreased more rapidly to a JADAS 10 of 11 at week 12 and further to 6.1 at week 24.

### Clinical examination

In total, 2970 separate joints of the hand region were evaluated by CE at three different points of time; of these, 2551 joints had complete data for US evaluation and 2910 had complete datasets for FOI analysis. Altogether, 519 of 2970 joints (17.5%) were clinically classified as active (434 (14.6%) swollen joints, 542 (18.2%) tender joints and 348 (11.7%) joints with limited range of motion). The joints PIP 2 and 3, followed by PIP 4 and 5, MCP 2 and 3 and the wrist were affected most frequently.

At baseline, 1110 joints were evaluated by CE and with FOI, and 990 joints were evaluated with US. In CE, 262 of the 1110 joints (23.6%) were classified as active joints (237 (21.4%) swollen joints, 267 (24.1%) tender joints and 181 (16.3%) joints with limited range of motion).

**Fig. 1:**
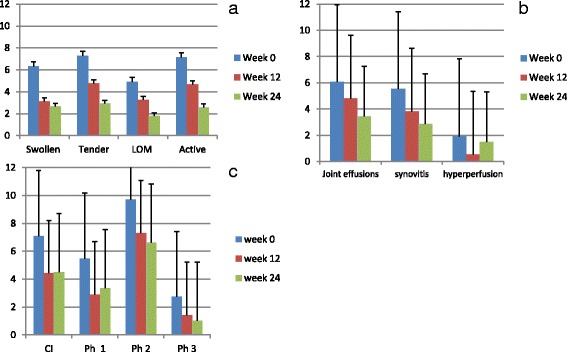
Mean affected joints of the hand region for the three examination techniques before the start of treatment and after 12 and 24 weeks of treatment. **a** Clinical examination. **b** US/PD examination. **c** Signal intensity on FOI examination. Mean values and standard deviations are shown. *LOM* limitation of motion, *CI* composite image, *Ph* phase

Upon treatment with either methotrexate or a biologic, a significant decrease of the mean number of active/swollen/tender or LOM joints at week 12 and week 24 compared with baseline was documented (Fig. 1a).

At week 12, 162 of 990 joints (16.4%) of the hand region evaluated by CE were assessed as clinically active. This decreased further in week 24 to 70 of 780 (9.0%).

### Ultrasonography findings

At baseline, 990 joints of the hand region were evaluated by US examination. Here 192 joints (19.4%) showed joint effusions, 186 joints (18.8%) showed signs of synovial thickening on GSUS and 68 joints (6.9%) showed signs of hyperperfusion on PDUS. These numbers had decreased at week 12 to 135 joints (16.1%) with joint effusions, 107 joints (12.7%) with synovial thickening and 15 joints (1.8%) with hyperperfusion of 840 joints of the hand region evaluated by US. At week 24, 720 joints of the hand region were assessed by US. While the numbers of joints with joint effusions and synovial thickening improved further (83 and 69 joints (11.5% and 9.6%) respectively), more joints showed signs of hyperperfusion compared with week 12 (36 joints (5%)).

At baseline, in total 243 joints (24.5%) showed signs of inflammation for any of the three US parameters, 161 joints (19.2%) at week 12 and 123 joints (17%) at week 24. As outlined in Fig. 1b, a decrease of the mean number of joints with effusion, synovial thickening and hyperperfusion at week 12 and week 24 compared with baseline was documented.

### Fluorescence optical findings

Overall, FOI revealed focal joint-related increased signal intensity as signs of synovitis/tenosynovitis in 947 joints. At baseline, 430 of 1110 evaluated joints (38.7%) showed increased signal intensity in one or more phases in FOI. This figure decreased to 280 of 960 evaluated joints (29.2%) at week 12 and 215 of 780 evaluated joints (27.6%) at week 24. With the phase1/phase 2/phase 3 and composite image, 215/369/108 and 266 joints were detected at baseline, 93/229/45 and 142 joints at week 12 and 84/166/26 and 115 joints at week 24 (Fig. 1c). Mean semiquantitative intensity of FOI signals in all examined joints decreased significantly between baseline and week 12 in all three phases and the composite image (*p* < 0.0001 for all), with only minimal changes between weeks 12 and 24.

**Fig. 2:**
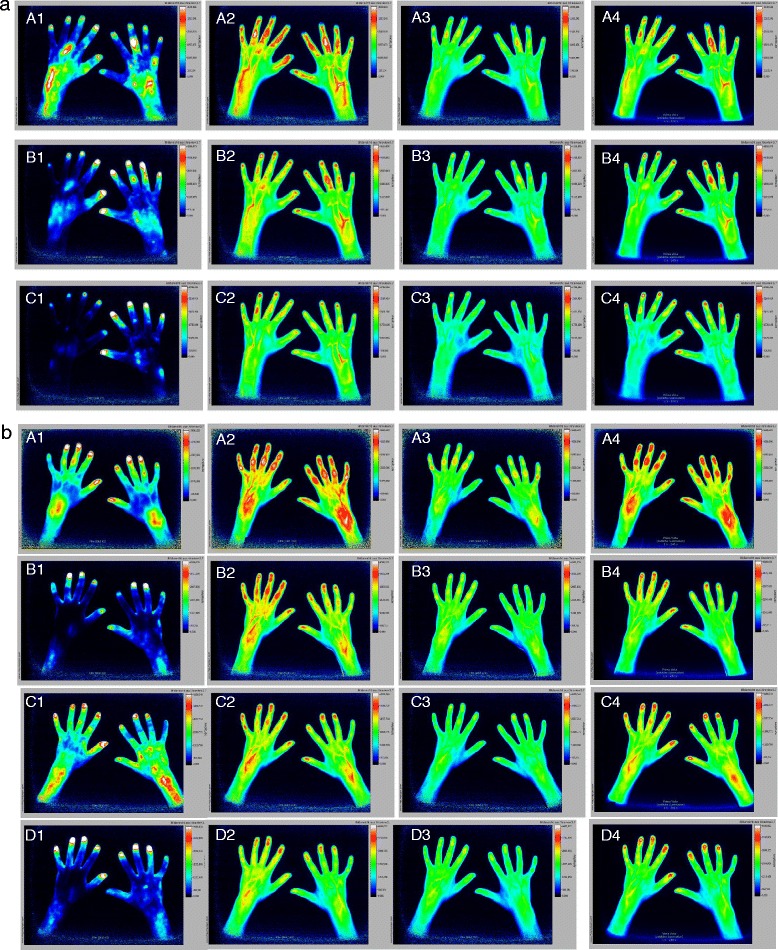
**a** FOI of a patient treated with adalimumab. *Row A*, baseline; *row B*, week 12; *row C*, week 24; *column 1*, phase 1; *column 2*, phase 2; *column 3*, phase 3; *column 4*, composite image. At baseline, signals detected were grade 3 over PIP 3 of the right hand in phases 1 and 2 and composite image. Left-hand PIP 2–5 showed grade 2 signals in phase 2 and grade 1 signal in phase 3 and composite image. Left-hand PIP 1 showed grade 2 in phase 2, grade 0 in phase 3 and grade 1 in composite image. After 12 weeks of treatment, improvement of signal intensity was visible. Especially, right-hand PIP 3 still shows grade 3 signal in phase 2. Further reduction of signaling was noted at week 24. **b** FOI of a patient with polyarticular JIA who started etanercept treatment. *Row A*, baseline; *row B*, week 12; *row C*, week 24; *row D*, week 52; *column 1*, phase 1; *column 2*, phase 2; *column 3*, phase 3; *column 4*, composite image. At baseline, both wrists, both side PIP 2–5 and DIP 2–4 showed grade 3 signals in phase 2 but grade 1 in phase 3. Both DIP 5 showed weaker signals. Twenty-four-week imaging, especially in phase 2, demonstrates subclinical activity. At clinical examination the patient already had no active joints. Upon constant clinical remission on treatment, there is further improvement of FOI findings visible after 1 year

Figure 2a shows FOI examinations of one patient at the three different time points. At baseline, high signals in FOI were noted, especially in PIP joints and in phase 2. These joints were also assessed active by clinical examination. All but one of the joints showed signs of effusions and most also synovial thickening on US, and PIP 3 of the right hand also showed hyperperfusion by PD. Upon treatment, the number of joints as well as the signal intensity decreased. At week 24, residual inflammation was visible by FOI and by clinical investigation especially in PIP 2–5 of the left hand.

Figure 2b shows FOI examinations of a second patient at four different time points. At baseline, both wrists and PIP 2–5 joints of both sides showed high signals in FOI, especially in phase 2 and in the composite image. In addition, signals over the DIP region indicate DIP joint involvement but cannot be clearly distinguished from fingertip hyperemia. Clinically DIP 2–5 of the left hand, MCP 1 of the left hand and PIP 2–5 of both hands were assessed as active, only PIP 2, 4 and 5 of the left hand showed sonographic effusions and PIP 5 of the left hand showed synovial thickening. Upon treatment, the number of joints as well as the signal intensity decreased. At week 24, residual inflammation was visible in the region of PIP joints and wrist, while clinically and with US/PD no joint was assessed as active. Thus, clinically the patient was in remission, showing residual inflammation in FOI only. This patient had an FOI examination 1 year after baseline following completion of the study, while reporting pain in DIP and wrist joints. Clinically there were no abnormal findings in joint status and US/PD. FOI showed only minor signal enhancement.

### Predictive value of FOI and clinical outcome

Joints showing improvement of FOI semiquantitative score at month 3 were more likely to improve clinically by month 6 than joints that stayed consistent or deteriorated with their FOI semiquantitative score (32–37% versus 18–20%). Logistic regression analysis, however, failed to produce reliable models. No positive correlation could be found between improvement of semiquantitative FOI score after 3 months and joint status after 6 months. Also no association between improvement in FOI scores of more than 30% at month 12 and JADAS minimal disease activity or JADAS remission could be shown.

### Comparison of FOI, US and CE

**Fig. 3:**
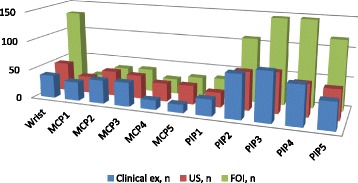
Clinical, US and FOI results on the single joint level (all points of time summarized). Clinical examination considered active joint definition. US considered any sign of effusion, synovial thickening or PD positivity. FOI considered signal enhancement in any phase. *ex* examination, *FOI* fluorescence optical imaging, *PIP* proximal interphalangeal joint, *MCP* metacarpophalangeal joint, *US* ultrasonography

The total number of joints evaluated for the hand region was 2970. Figure 3 summarizes CE, FOI and US results on the single joint level, showing the differences between the joints concerning frequency of detection of pathology by examination technique. FOI detected the highest number of joints (947 joints, 32%). Abnormal US findings were present in 527 of 2550 joints (20.7%) evaluated by US. A total 519 of the evaluated joints (17.5%) had active arthritis CE. A high number of joints (627 joints, 66.2%) showing increased signal intensity by FOI were rated clinically inactive by CE. Of the joints rated active in CE, 61.7% also showed FOI increased signal intensity. A total of 199 joints with clinical abnormalities did not show signaling by FOI (Table 2).

**Table 2:** Number of joints from a total of 2970 evaluated joints at three different points of time and comparison of number of joints with increased FOI signal and detection by US according to detection by clinical examination

	FOI					US/PD			
	Any signal (*n* = 947)	Composite image (*n* = 530)	Phase 1 (*n* = 396)	Phase 2 (*n* = 783)	Phase 3 (*n* = 180)	Any signal (*n* = 527)	Effusion (*n* = 430)	Synovitis (*n* = 375)	Hyperperfusion (*n* = 120)
Signal in clinically active joints (*n* = 519)	320 (33.8%/61.7%)^a^	205 (38.7%/39.5%)	161 (40.7%/31.0%)	277 (35.4%/53.4%)	81 (45.0%/15.6%)	239 (45.4%/46.0%)	211 (49.1%/40.7%)	164 (43.7%/31.6%)	47 (39.2%/9.0%)
Signal in clinically inactive joints (*n* = 2451)	627 (66.2%/25.6%)	325 (61.3%/13.3%)	235 (59.3%/9.6%9)	506 (64.6%/20.6%)	99 (55.0%/4.0%)	288 (54.6%/11.8%)	219 (51.0%/8.9%)	211 (56.2%/8.6%)	73 (60.8%/3.0%)

Data on US examination were available for 2550 joints in total

*FOI* fluorescence optical imaging, *PD* Power Doppler mode, *US* ultrasonography

^a^Percentages refer to columns/rows

Also 513 (61.7%) joints that did not show sonographic signs of synovial thickening, effusions or hyperperfusion were detected by FOI. Especially, phase 2 showed increased signal intensity more often than the other phases and composite image.

On US examination, 211 of the 519 (40.6%) clinically active joints over all three time points showed effusion, 164 (31.6%) synovial thickening and 47 (9%) signs of hyperperfusion.

Ultrasonography did not detect signs of effusion, signs of synovial thickening or signs of hyperperfusion in numerous joints that were clinically classified as active. Effusions and synovial thickening were detected by US in 219 (8.9%) and 211 (8.6%) of 2451 clinically inactive joints and hyperperfusion was detected by power Doppler in 73 (3.0%) clinically inactive joints (Table 2).

Agreement of FOI with US was analyzed with obvious differences between different joints and in the different phases of FOI. Interestingly, agreement was 75–100% in DIP joints and MCP 1 (phase 2, CI), was 64–92% (CI) and 72–91% (phase 2) for MCP 2–5 and was 41–63% (CI) and 38–69% (phase 2) for PIP 2–4. A high variance between patients was also detected: mean agreement over patients (for all joints) ranged from 33 to 100% with a width of the confidence interval from 16 to 21%, depending on the compared pair of analysis methods.

FOI in phase 2 agreed best with detection of US effusions and synovial thickening, while the detection of PDUS hyperperfusion correlated best with FOI in phase 1 and to a slightly lesser extent phase 2.

Specificity of FOI compared with CE and US as the standard of reference showed high percentages for all parameters. Comparison of FOI phase 2 with clinical and US parameters showed specificity between 73 and 80%, composite image 82–87% and the other phases around or over 90%. Comparison of US parameters of inflammation and CE as standard of reference specificity was between 86 and 97% (Table 3).

**Table 3:** Sensitivities: comparison of FOI parameters and parameters of clinical examination and US parameters, and comparison of US parameters and clinical examination parameters

Reference	FOI				US		
	Composite image	Phase 1	Phase 2	Phase 3	Joint effusion	Synovitis	Hyperperfusion power Doppler
Clinical active joints	0.395	0.314	0.543	0.160	0.471	0.360	0.103
Clinical swollen joints	0.394	0.323	0.534	0.169	0.474	0.365	0.103
Clinical tender joints	0.315	0.237	0.442	0.108	0.306	0.269	0.068
LOM	0.481	0.423	0.664	0.209	0.497	0.440	0.173
US joint effusion	0.393	0.316	0.565	0.191			
US synovitis	0.358	0.358	0.522	0.139			
Hyperperfusion by power Doppler	0.306	0.604	0.477	0.126			

*FOI* fluorescence optical imaging, *LOM* limitation of motion, *US* ultrasonography

Sensitivities comparing FOI and US with CE as the standard of reference as well as comparing FOI with US as the standard of reference were lower, with FOI phase 2 delivering the best values (45–56%) (Table 4).

**Table 4:** Specificities: comparison of FOI parameters and parameters of clinical examination and US parameters, and comparison of US parameters and clinical examination parameters

Reference	FOI				US		
	Composite image	Phase 1	Phase 2	Phase 3	Joint effusion	Synovitis	Hyperperfusion power Doppler
Clinical active joints	0.865	0.902	0.791	0.958	0.897	0.906	0.969
Clinical swollen joints	0.856	0.896	0.777	0.955	0.887	0.899	0.967
Clinical tender joints	0.848	0.884	0.767	0.947	0.862	0.888	0.962
LOM	0.859	0.903	0.785	0.957	0.874	0.899	0.974
US joint effusion	0.856	0.909	0.784	0.959			
US synovitis	0.841	0.904	0.762	0.945			
Hyperperfusion by power Doppler	0.818	0.888	0.730	0.935			

*FOI* fluorescence optical imaging, *LOM* limitation of motion, *US* ultrasonography

**Fig. 4:**
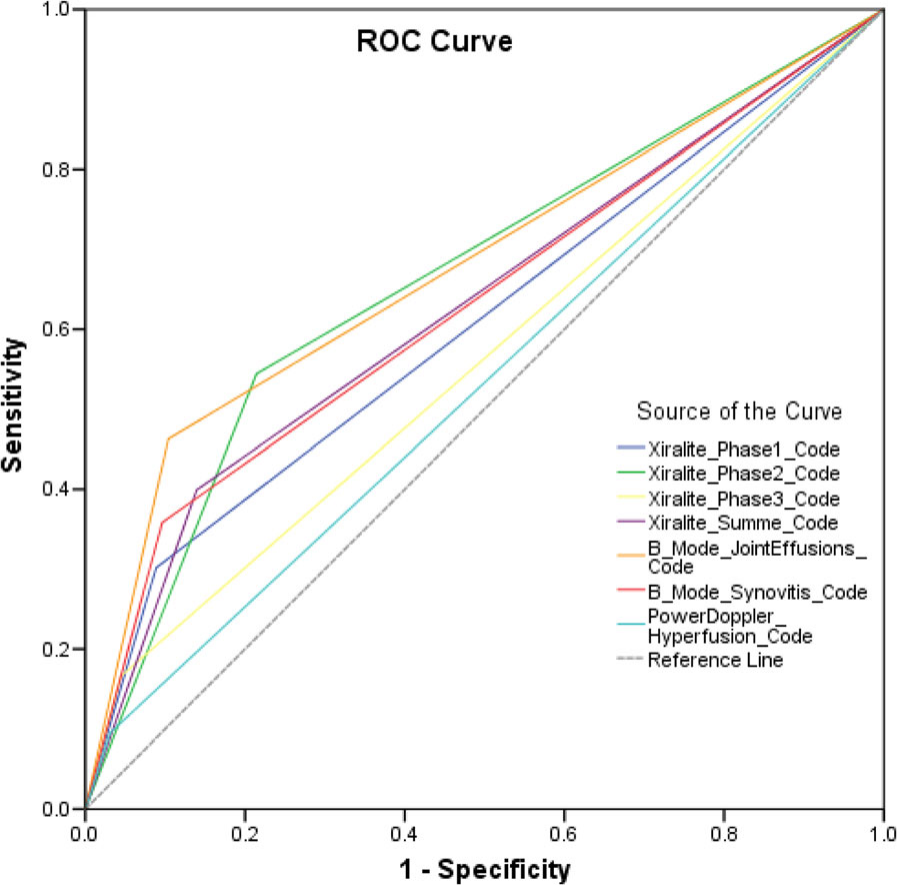
ROC curves for FOI parameters in phases 1–3 and CI and US parameter B-mode joint effusion, B-mode synovitis and PDUS compared with clinical examination (active joint). Area under the curve (95% CI): FOI phase 1, 0.61 (0.58–0.64); FOI phase 2, 0.67 (0.64–0.70); FOI phase 3, 0.56 (0.53–0.59); FOI CI, 0.63 (0.60–0.66); US joint effusion, 0.68 (0.65–0.71); US synovitis, 0.63 (0.60–0.66); PDUS, 0.53 (0.50–0.56). *ROC* Receiver operating characteristic

**Fig. 5:**
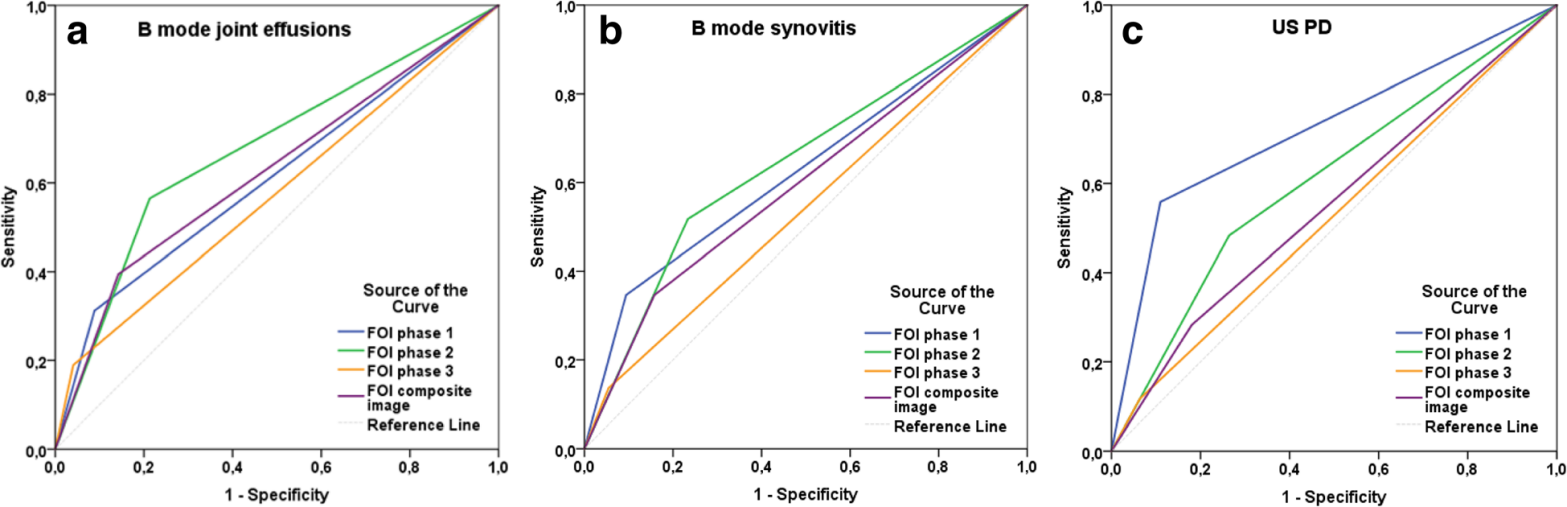
ROC curves for FOI parameters in phases 1–3 and CI compared with US parameters B-mode joint effusion, B-mode synovitis and PD hyperperfusion. **a** US parameter B-mode joint effusion (area under the curve (95% CI)): FOI phase 1, 0.61 (0.58–0.64); FOI phase 2, 0.68 (0.65–0.71); FOI phase 3, 0.57 (0.54–0.61); FOI CI, 0.63 (0.60–0.66). **b** US parameter B-mode synovitis (area under the curve (95% CI)): FOI phase 1, 0.63 (0.59–0.66); FOI phase 2, 0.64 (0.61–0.67); FOI phase 3, 0.54 (0.51–0.57); FOI CI, 0.59 (0.56–0.63). **c** US parameter PD hyperperfusion (area under the curve (95% CI)): FOI phase 1, 0.72 (0.67–0.78); FOI phase 2, 0.61 (0.56–0.66); FOI phase 3, 0.53 (0.47–0.58); FOI CI, 0.55 (0.50-0.61). *ROC* Receiver operating characteristic

ROC curves comparing FOI and US versus CE as the standard of care showed the highest values of the AUC for FOI phase 2 (AUC = 0.67 (95% CI 0.64–0.70)) and US B-mode joint effusions (AUC = 0.68 (95% CI 0.65–0.71)), followed by FOI composite image (AUC 0.63 (95% CI 0.60–0.66)) and US B-mode synovitis (AUC = 0.63 (95% CI 0.60–0.66)) (Fig. 4). Comparing FOI with US B-mode parameter joint effusions, phase 2 (AUC = 0.68 (95% CI 0.65–0.71)) followed by composite image (AUC = 0.63 (95% CI 0.60–0.66)) had the highest AUC values. Comparing FOI with US B-mode parameter synovitis, FOI phase 2 (AUC = 0.64 (95% CI 0.61–0.67)) and FOI phase 1 (AUC = 0.63 (95% CI 0.59–0.66)) had the highest AUC values. When calculating AUC with PDUS as the reference, FOI phase 1 had the highest values (AUC = 0.72 (95% CI 0.67–0.78)) (Figs. 5 a–c).

### Safety

During the 24 weeks of this study, 15 adverse events (AE) were reported in 10 different patients. These included six infectious events, four events with gastrointestinal complaints, three patients with flare of JIA and two intraarticular injections. None of the events were serious. No events in correlation to FOI examination were noted.

## Discussion

Musculoskeletal ultrasonography (in GSUS and PDUS) provides an imaging method which has a solid component in the clarification of inflammatory joint diseases. A disadvantage of the method is the relatively strong investigator dependency [18].

Morphological features of the inflammation are hypervascularization, increased blood perfusion and capillary permeability [19]. The (neo)angiogenesis is a very early event in the pathogenesis of chronic inflammation [20]. The angiogenesis of the synovial membrane leads to a dense capillary network in the inflamed joint membrane. Vasodilatation and increased capillary permeability maintain synovitis.

It has been shown in rheumatoid arthritis patients that the synovial vascularization correlates with the disease activity of an affected joint [21]. Arthrosonographic procedures, including power Doppler sonography, are limited in resolution, however, and are not capable of displaying an increased blood flow in the submillimeter region and thus on the capillary plane.

Magnetic resonance imaging (MRI) is today regarded as the gold standard for the representation of synovitis. Furthermore, bone marrow edema detectable in MRI is the strongest independent predictor of the radiological progression of rheumatoid arthritis [22, 23]. The exact significance of MRI for the diagnosis and prognosis of an early arthritis has not yet been clarified conclusively [24]. Overall, MRI is used for early diagnosis in clinical routine and above all in clinical trials. For standardization, the OMERACT criteria for the use and evaluation of MRI were developed [25]. However, there are limitations to the use of MRI in everyday practice due to the high cost of an examination and the limited availability. The use of gadolinium and some contraindications also limit their use in clinical routine. Furthermore, there were several limitations especially in younger children, such as maintaining an immobile position or the necessity for sedation.

In human medicine, FOI methods have so far been used predominantly in ophthalmology as fluorescence angiography for the imaging diagnostics of retinal and choroidal diseases. Furthermore, FOI use is described in vascular surgery, in neurosurgery after clipping of a brain aneurysm in oncology to delineate tumor tissue and in plastic and reconstructive surgery [9, 26, 27].

FOI is a relatively new imaging technique for the detection of inflammation and arthritis in the joints of the hand by visualizing altered microcirculation and angiogenesis in adults with rheumatoid arthritis [9, 28]. FOI findings agreed well with US [10, 11] and MRI [11] findings in two studies with 252 and 32 RA patients and 46 and 6 patients respectively without signs of inflammatory disease as control groups. Early arthritis as well as subclinical inflammation could be detected by FOI.

There are almost no data concerning FOI use in pediatric patients with JIA. Werner et al. [29] described FOI use and results in three children. The aim of this study was to evaluate patients with JIA of hand and finger joints using the FOI technique along with US and CE, and to show its usefulness for demonstration of improvement upon treatment. Thus a follow-up analysis before and after 12 and 24 weeks of treatment (either methotrexate or a biologic antirheumatic drug) using the FOI technique in comparison with US and CE was performed in an open-label study. Clinical findings were all as expected. A high rate of treatment success on either a newly initiated therapy with methotrexate or a biologic was documented, similar to that observed in other trials, with earlier improvement in the biologics cohort.

As one result of our study, the number of joints with increased signal intensity in FOI was higher than the number of joints rated clinically active and even higher than the joints with signs of inflammation on US. These findings are consistent with the results of former studies using FOI in adults [10, 11]. In comparative studies of CE and GSUS, PDUS, and MRI, inflammatory changes were documented in clinically asymptomatic joints [30–32]. It can be assumed that FOI detected clinical and also subclinical activity.

There were differences in the specific phases of the analysis. The interpretation of the different phases is still not clear. Especially, phase 2 showed increased signal intensity in more joints than any other phase, including the composite image. Comparison of FOI with CE showed that phase 2 was best at detecting clinical active joints and also showed more activity in clinical inactive joints compared with the other FOI phases. FOI phase 2 also agreed best with US signs of synovial thickening and effusions. This may be the most sensitive phase and may be best suited to detect subclinical activity.

Phase 3 rarely showed increased FOI signals. In the former studies in adults, the authors considered phase 3 to show increased capillary permeability in which ICG is more persistent than normal [10, 11].

In joints with hyperperfusion detected by PDUS, FOI phase 1 most often showed increased signal intensity, followed by phase 2. This can also be shown in the ROC curves; when comparing the different FOI phases with PDUS, FOI phase 1 reached the best AUC values. As proposed by studies in adult patients, phase 1 may mirror increased vascularity as a sign of high disease activity [10, 11].

With CE as the standard of reference, FOI and US showed moderate sensitivities and high specificities, partly due to the high number of negative results. The findings for sensitivity and specificity for FOI with US as the standard of reference were comparable. This could also be shown in the ROC curves. Compared with CE, FOI phase 2 and US B-mode joint effusions were the most accurate parameters with similar values for the AUC. FOI phase 2 also had the highest AUC values when compared with the US mode parameters of joint effusion and synovitis. Because of the lack of an assured standard of reference, the interpretation of sensitivity and specificity is a delicate issue. Of course the majority of examined joints were expected to be inactive. Specificity was influenced by higher rates of positive findings in FOI compared with the other two methods. Because of the lack of a control group and an assured standard of reference like histopathological findings, sensitivity and specificity should be valued carefully. Results for agreement were added to complete the diagnostic description, but it should be considered that high numbers are partly based on the high amount of negative results.

In the detection of inflammatory changes, FOI was more sensitive than CE. The higher rate of positive findings compared with GDUS/PDUS suggests that the method is more sensitive than the other two procedures. It could be argued that only mild increases in FOI signal intensity might not be a sign of active arthritis and discreet findings in FOI should be interpreted carefully. Only FOI signals attributable to and limited to a specific joint were rated as FOI-positive joints. Also, Werner et al. [10, 11] state in their former studies that, because healthy subjects and controls with arthralgia but without signs of inflammatory disease had virtually no pathological increased signal intensity in FOI using the Xiralite method, the detected FOI signals appear to reflect actual inflammatory activity and are not all to be interpreted as false-positive findings.

Using MRI as a standard reference method, ICG-assisted fluorescence-optical diagnostics demonstrated high sensitivity (up to 85%) and high specificity (up to 95%) for the detection of MRI synovitis and tenosynovitis, as well as high consistency rates with MRI [10]. Unfortunately we were not able to also include MRI analyses in our study.

Improvement after 12 and 24 weeks of antirheumatic treatment could be shown with all three techniques, while the decrease in the number of joints with a FOI signal was slower than the clinical changes. To a lesser extent this was seen for the US as well. This indicates that joints becoming clinically inactive with treatment may show undetected subclinical activity, even after months of treatment. This is obvious in our patient shown in Fig. 3b who had residual inflammation at week 24 demonstrated by FOI although being in clinical remission. Hence, FOI can be useful to assess treatment response.

FOI improvement at month 3 did not show reliable predictive value for clinical improvement at month 6. Although more joints had changed from active at month 0 to inactive at month 6, when they had improved in FOI semiquantitative scores at month 3 (all FOI phases) the difference from 19 to about 35% is not huge. Logistic regression analysis could not produce reliable models either. One problem might be differences in sample size, because the vast majority of joints were inactive in the first place and did not show clinical or FOI changes during the investigation. But calculating only with initially active joints did not change the probability of outcome prediction.

There were some limitations to our study. The mean age of our patient cohort was relatively high, the youngest patient being 9 years old. This was due to reduced compliance in younger patients because the methodology is limited to older children able to comply with the procedure which takes 6 minutes and to the arm length of the children.

One further limitation of the FOI method is the lack of visualization of anatomical structures such as synovial proliferation or erosion, as well as the limited ability to assess the palmar inflammation, especially in the area of the hand and MCP joints. Furthermore, any inflammation of the hand region, for example lacerations of the skin, will result in increased signal intensity. In this study all patient hands were also examined for signs of inflammation and injury other than arthritis; this was documented and visible findings were documented and considered for FOI reading. Finally, FOI examination was tolerated well, with no report of AE correlated with the examination. We did not observe any serious adverse events in our cohort and in no patients did the analysis have to be stopped or interrupted.

## Conclusion

ICG-supported FOI with the Xiralite method is a new imaging method for rheumatological questions. This technology allows sensitive detection of inflammatory changes. It is a useful technique to supplement clinical and ultrasonographic investigation in assessment of JIA activity in the joints of the hand. It can provide useful additional information for diagnosis and assessment of treatment response in JIA. Subclinical activity probably can be detected with this method. The procedure could be particularly important for the diagnosis of early arthritis as well as for follow-up under long-term anti-inflammatory therapy or for evaluation of remission.

## Abbreviations

AUC: Area under the curve
CE: Clinical examination
CHAQ: Childhood Health Assessment Questionnaire
CI: Composite image
CRP: C-reactive protein
DIP: Distal interphalangeal joint
DMARD: Disease-modifying antirheumatic drug
ESR: Erythrocyte sedimentation rate
FOI: Fluorescence optical imaging
GS: Grey-scale mode
ILAR: International League of Rheumatology
ITT: Intention to treat
JADAS: Juvenile arthritis disease activity score
JIA: Juvenile idiopathic arthritis
LOM: Limitation of motion
MCP: Metacarpophalangeal joint
MRI: Magnetic resonance imaging
MTX: Methotrexate
NRS: Numeric rating scale
OMERACT: Outcome Measures in Rheumatology
PD: Power Doppler mode
PedACR: American College of Rheumatology pediatric criteria
PIP: Proximal interphalangeal joint
ROC: Receiver operating characteristic
US: Ultrasonography
